# Exploring the impact of design criteria for reference sets on performance evaluation of signal detection algorithms: The case of drug–drug interactions

**DOI:** 10.1002/pds.5609

**Published:** 2023-03-26

**Authors:** Elpida Kontsioti, Simon Maskell, Munir Pirmohamed

**Affiliations:** ^1^ Department of Electrical Engineering and Electronics University of Liverpool Liverpool UK; ^2^ The Wolfson Center for Personalized Medicine, Center for Drug Safety Science, Department of Pharmacology and Therapeutics Institute of Systems, Molecular and Integrative Biology, University of Liverpool Liverpool UK

**Keywords:** adverse events, drug–drug interactions, performance metrics, pharmacovigilance, postmarketing surveillance, signal detection, spontaneous reports data

## Abstract

**Purpose:**

To evaluate the impact of multiple design criteria for reference sets that are used to quantitatively assess the performance of pharmacovigilance signal detection algorithms (SDAs) for drug–drug interactions (DDIs).

**Methods:**

Starting from a large and diversified reference set for two‐way DDIs, we generated custom‐made reference sets of various sizes considering multiple design criteria (e.g., adverse event background prevalence). We assessed differences observed in the performance metrics of three SDAs when applied to FDA Adverse Event Reporting System (FAERS) data.

**Results:**

For some design criteria, the impact on the performance metrics was neglectable for the different SDAs (e.g., theoretical evidence associated with positive controls), while others (e.g., restriction to designated medical events, event background prevalence) seemed to have opposing and effects of different sizes on the Area Under the Curve (AUC) and positive predictive value (PPV) estimates.

**Conclusions:**

The relative composition of reference sets can significantly impact the evaluation metrics, potentially altering the conclusions regarding which methodologies are perceived to perform best. We therefore need to carefully consider the selection of controls to avoid misinterpretation of signals triggered by confounding factors rather than true associations as well as adding biases to our evaluation by “favoring” some algorithms while penalizing others.

**Key Points**

- Performance assessment of SDAs in pharmacovigilance has often relied on the generation of custom‐made reference sets of limited size that consider ad‐hoc exclusion or inclusion criteria to define eligible controls.
- SDA performance assessment might be biased based on the selected benchmarks, as each methodology can be impacted to a different extent by different confounders.
- We tested 14 design criteria for reference sets in the case of DDIs, showing that some of them considerably affected the performance and comparative evaluation of different SDAs for DDI surveillance while others did not have a significant effect.
- Overall, this analysis advocates the utilization of large, to the extent possible, reference sets that are less likely to suffer from overrepresentation of controls that make different SDAs behave in different ways due to confounding. Any decision to restrict the evaluation set using specific design criteria should be carefully justified.

**Plain Language Summary**

Reporting of suspected side effects experienced by patients following drug approval is a key component to identifying novel drug safety issues. Statistical methods are then used to analyze reports and reveal signals of novel associations between drugs and side effects. Performance evaluation of those methods traditionally relies on custom‐made reference sets of limited size that consider ad‐hoc exclusion or inclusion criteria to define eligible controls. However, each method can be impacted to a different extent by those criteria, as they can act as potential confounders. This study investigated the impact of 14 criteria on three methods that have been developed to detect signals of potential adverse drug–drug interactions, showing that some of them had opposing effects or effects of different levels of magnitude on the performance of the different methods. The relative composition of reference sets can therefore significantly affect the evaluation metrics, potentially altering the conclusions regarding which methodologies are perceived to perform best. The selection of controls should be carefully performed to avoid misinterpretation of signals triggered by confounding factors rather than true associations as well as adding biases to our evaluation by “favoring” some algorithms while penalizing others.

## 1 INTRODUCTION

Monitoring drug safety issues during the post‐approval phase requires reporting of suspected drug‐related adverse reactions by healthcare professionals, patients, and pharmaceutical companies. The reports are collected in spontaneous reporting system (SRS) databases, such as the FDA Adverse Event Reporting System (FAERS) database in the US, the Eudravigilance database in the EU, and the Yellow card database in the UK. These databases form an important part of the pharmacovigilance strategy since they not only contain information on adverse events (AEs) and suspected drugs, but also details regarding concomitant medications, indications, and patient demographics.

By applying statistical methods known as signal detection algorithms (SDAs), novel associations between drugs and AEs (i.e., signals) that have not been identified in clinical trials can be identified in the SRS data. Given the absence of a control group, SDAs predominantly rely on disproportionality analysis, which calculates the degree of disproportional reporting of drug‐AE combinations compared to what would be expected if there were no association between them.^1^ However, the presence of synthetic associations (i.e., causative covariates that have not been taken into account or remain unobserved) can lead to confounding, either upward or downward, thus generating faulty associations between the drug and the AE and complicating the detection of safety signals.^2^, ^3^, ^4^ For example, reporting quality issues arising from a poor distinction between symptoms of disease‐related AEs and treatment effects of drugs (or drug combinations) is a result of a synthetic association called confounding by indication.^5^, ^6^

The practice of using larger clusters of medical terms to perform quantitative signal detection in pharmacovigilance has been widely discussed in the literature.^1^, ^7^ Many previous efforts investigated the impact of the Medical Dictionary for Regulatory Activities (MedDRA) granularity on signal detection tasks.^8^, ^9^ Also, many studies have considered the use of term grouping to identify relevant reports.^10^, ^11^ However, recommendations from the IMI‐PROTECT project suggest that signal detection at the PT level should be considered the standard approach in real‐life pharmacovigilance.^9^, ^12^

The development of novel SDAs in pharmacovigilance requires the existence of appropriate reference sets that can be utilized both for absolute performance evaluation as well as for comparison with existing methodologies. Given that each SDA, depending on the applied modeling, might be impacted to a different extent by a confounder, the performance evaluation might be biased based on the selected benchmarks. The challenge of building appropriate reference sets in pharmacovigilance has been previously acknowledged in the literature.^13^, ^14^, ^15^, ^16^ Most studies have attempted to comparatively evaluate SDAs by testing their performance against custom‐made reference sets, often limited in size^17^, ^18^, ^19^ or not publicly available^20^, ^21^ which commonly consider ad‐hoc inclusion or exclusion criteria to generate positive and negative controls. Examples of such criteria include those related to AE background prevalence (given that, in disproportionality analysis, the denominator signifies the expected rate of occurrence),^22^ disease‐related AEs,^23^ AE seriousness^23^, ^24^ or evidence associated with positive controls.^22^, ^23^, ^24^, ^25^, ^26^ The criteria are typically used to attempt to address the limitations of disproportionality analysis and to tackle issues with potential confounders.

In the case of adverse drug–drug interactions (DDIs), signal detection is considered more complicated, with the existing methodology being less mature compared to the one in the case of signals for single drugs. A previous study has suggested that the detection of DDI‐related signals might suffer from multiple confounders.^27^ For example, concomitant medications appear to be a significant source of confounding (i.e., the signal associated with a drug combination triggered by drugs that are usually given concomitantly but not signifying true adverse drug–drug‐event associations). In addition, only limited efforts exist in the literature to generate reference sets related to two‐way DDIs.^17^, ^19^, ^27^, ^28^

In this study, we aim to explore the relative impact of different factors that could be potential sources of confounding on the performance evaluation of existing methods for signal detection of DDIs. By utilizing a large and diversified reference set, we were able to create custom‐made reference sets considering multiple design criteria to assess any differences observed in the quantitative evaluation of SDAs tailored for two‐way DDIs.

## 2 METHODS

### 2.1 Data sources

#### 2.1.1 FAERS data—spontaneous reports

We used a curated and standardized version of the publicly available FAERS database. The data pre‐processing pipeline was based on the Adverse Event Open Learning through Universal Standardization (AEOLUS) process and included removal of duplicate reports, drug name normalization at the RxNorm ingredient level, and AE mapping to MedDRA Preferred Terms (PTs).^29^ The curated data set included 9 203 239 reports containing at least one drug and one AE between 2004 (Q1) and 2018 (Q4), with 3 973 749 (43.18%) reports mentioning more than one drug. Each drug was considered equivalent in the analysis irrespective of its reported role (i.e., primary suspect; secondary suspect; concomitant; and interacting).

#### 2.1.2 Reference sets for DDIs

CRESCENDDI, a reference set for two‐way DDIs, was the primary source of controls.^30^ This reference sets covers 454 drugs and 179 adverse events mapped to RxNorm Ingredient and MedDRA PT concepts, respectively, from the Observational Medical Outcomes Partnership (OMOP) Common Data Model (version 5). We used 4455 positive and 4544 negative controls from CRESCENDDI that were also present in the curated FAERS dataset (hereafter called PT Reference Set).

To accommodate and test the impact of MedDRA granularity to detect signals at the medical concept (MC) level, we extended CRESCENDDI by building PT groups (event groups), where possible, that are relevant to the adverse events described in the original reference set. These groups were formed by examining Standardized MedDRA Queries (SMQs) and event definitions from a time‐indexed reference standard by Harpaz et al.^31^ and were manually reviewed for clinical relevance. In total, 20 adverse events from CRESCENDDI were deemed suitable for extension to the MC level (Table 1). A full list of the event groups is available in Appendix S1. The new reference set (hereafter called MC Reference Set) contained 1097 positive and 614 negative controls (Appendix S2).

**TABLE 1:** Medical concepts in the MC Reference Set.

Name		
Acute kidney injury	Drug‐induced liver injury	Myopathy
Acute psychosis	Hyperglycemia	Priapism
Angioedema	Hypertension	Rhabdomyolysis
Arrhythmia	Hypoglycemia	Tachycardia
Bradycardia	Hypernatremia	Thrombocytopenia
Cardiac failure	Hypothyroidism	Torsade de pointes
Drug withdrawal syndrome	Lactic acidosis	

### 2.2 Data mining

We performed the case/non‐case analysis at two different levels, based on the reference sets that we utilized. The first one was restricted to the reports that included the PT that was related to each control from the PT Reference Set. The second one considered as cases all the reports that contained any of the PTs that were part of the MC linked to the control in the MC Reference Set.

For example, the case/non‐case analysis for a control related to torsade de pointes resulted in two contingency tables: the first one only considered the PT “Torsade de pointes” to retrieve case reports, while the second one included the following terms (as PTs): “Electrocardiogram QT interval abnormal”, “Electrocardiogram QT prolonged”, “Long QT syndrome”, “Torsade de pointes”, “Ventricular tachycardia”. Non‐cases included the reports without the aforementioned PTs, while reports containing more than one of the relevant PTs linked to the MC were not double‐counted.

### 2.3 Design criteria

Table 2 shows the design criteria that were considered as potential confounding factors, which fall into the following categories: (i) evidence level; (ii) event seriousness; (iii) event frequency; (iv) potential confounding by indication; and (v) potential confounding by concomitant medication. PT Reference Set controls were stratified based on each of the design criteria, forming suitable restricted subsets of different sizes in each case, depending on the criterion under consideration. MC Reference Set could not be stratified using categories (ii) and (iii).

**TABLE 2:** Categories and descriptions of design criteria for reference sets that could affect performance evaluation of SDAs for DDI surveillance.

Category	Design criterion (DC)	Description
Evidence level	BNF—Study	Interactions where the information is based on formal study including those for other drugs with the same mechanism, for example, known inducers, inhibitors, or substrates of cytochrome P450 isoenzymes or *P*‐glycoprotein.
	BNF—Theoretical	Interactions that are predicted based on sound theoretical considerations. The information may have been derived from in vitro studies or based on the way other members of the same class act.
	BNF—Anecdotal	Interactions based on either a single case report or a limited number of case reports.
	Micromedex—Established	Controlled studies have clearly established the existence of the interaction.
	Micromedex—Theoretical	The available documentation is poor, but pharmacologic considerations lead clinicians to suspect the interaction exists; or documentation is good for a pharmacologically similar drug.
	Micromedex—Probable	Documentation strongly suggests that the interactions exist, but well‐controlled studies are lacking.
Event seriousness*	EMA Important Medical Event (IME) Terms	Any untoward medical occurrence that at any dose: * results in death, * is life‐threatening, * requires inpatient hospitalization or prolongation of existing hospitalization, * results in persistent or significant disability/incapacity, or * is a congenital anomaly/birth defect.
	EMA Designated Medical Event (DME) Terms	Medical conditions that are inherently serious and often medicine‐related (e.g., Stevens‐Johnson syndrome). This list does not address product‐specific issues or medical conditions with high prevalence in the general population.
Event frequency*	Common PTs	PT prevalence ≥90th percentile of the prevalence of PTs reported in FAERS
	Rare PTs	PT prevalence ≤10th percentile of the prevalence of PTs reported in FAERS
Potential confounding by indication	AE is an indication–True	The AE is also an indication for at least one of the two drugs from the drug–drug‐event triplet under consideration
	AE is an indication—False	The AE is not an indication for either of the drugs from the drug–drug‐event triplet under consideration
Potential confounding by concomitant medication	Shared indications—False	Drug pairs that share at least one indication are excluded
	Shared indications—True	Only drug pairs that share at least one indication are considered

*Note*: The categories marked with an asterisk (*) contain design criteria that were not applicable to the MC Reference Set.

### 2.4 PT prevalence

The impact of reference set restriction by PT prevalence on the Area Under the Curve (AUC) estimates was also examined. The PT prevalence was calculated in the curated FAERS data set as the frequency of PTs from reports containing at least one drug. We grouped the 179 PTs from the PT Reference Set using quartile binning of their prevalence. The controls were then stratified into four groups (Groups Q1–Q4) based on their PTs by considering the respective PT prevalence quartile.

### 2.5 SDAs

Three SDAs that have been previously described in the literature were considered:

- An observed‐to‐expected shrunk interaction measure (Omega)^32^;
- The “interaction coefficient” in a linear regression model with additive baseline (delta_add)^33^;
- A measure based on an adapted version of Multi‐Gamma Poisson Shrinker (MGPS) model, called Interaction Signal Score (IntSS).^17^

### 2.6 Impact of MedDRA granularity on SDA performance evaluation

To assess the impact of MedDRA granularity on the SDAs that were considered in this study, we performed a Receiver Operating Characteristic (ROC) analysis to examine the difference in AUC when considering matched controls from the two reference sets.

### 2.7 Estimation of design criteria impact on SDA performance evaluation

For each reference set and design criterion, we simulated the generation of a constrained reference set by randomly drawing an equal number (1:1) of positive and negative controls from the restricted control subset that used the specified design criterion for control stratification. An unconstrained reference set of equal size was generated in each case by following a similar process but using the original reference set. This sampling generation process took into account the correlation between the two sets, as the probability of drawing one control for the constrained reference set did not affect the probability of drawing any control for the unconstrained reference set. The size of the simulated reference sets varied from 100 to $2\times N_{\max}$, where $N_{\max}$ was determined by either the number of positive or negative controls (depending on which one was smaller) in each of the restricted subsets. For each SDA, we calculated: (i) AUC scores; and (ii) positive predictive value (PPV) for fixed sensitivity values (i.e., 0.60, 0.75, and 0.90) for both reference set types (i.e., constrained and unconstrained) by performing 1000 simulations. The statistics of the samples were summarized by fitting a Normal distribution, for which we report the mean and variance. The difference of the means of AUC (${\mathrm{AUC}}_{\text{diff}}$), and PPV (${\mathrm{PPV}}_{\text{diff}}$) (with 95% confidence intervals) were the target measures. The probability of ${\mathrm{AUC}}_{\text{diff}}$ being non‐zero, $P\left(|{\mathrm{AUC}}_{\text{diff}}|>0\right)$, was also estimated under the normality assumption:

(1) $$\begin{array}{l} \left|{\mathrm{AUC}}_{\text{diff}}\right|\sim N(\left|\mu_{{\mathrm{AUC}}_{\text{Restricted}\_\mathrm{ROC}}}–\mu_{{\mathrm{AUC}}_{\text{Unrestricted}\_\mathrm{ROC}}}\right|,\\ \;\sqrt{{\sigma_{{\mathrm{AUC}}_{\text{Restricted}\_\mathrm{ROC}}}}^{2}+{\sigma_{{\mathrm{AUC}}_{\text{Unrestricted}\_\mathrm{ROC}}}}^{2}\;}) \end{array}$$

(2) $$P\left(\left|{\mathrm{AUC}}_{\text{diff}}\right|>0\right)=1-P\left(\left|{\mathrm{AUC}}_{\text{diff}}\right|=0\right)=1–F_{{\mathrm{AUC}}_{\text{diff}}}\left(0\right)$$

where μ is the mean, σ is the standard deviation, and $F_{{\mathrm{AUC}}_{\text{diff}}}$ is the normal cumulative distribution function (CDF) of ${\mathrm{AUC}}_{\text{diff}}$.

**FIGURE 1:**
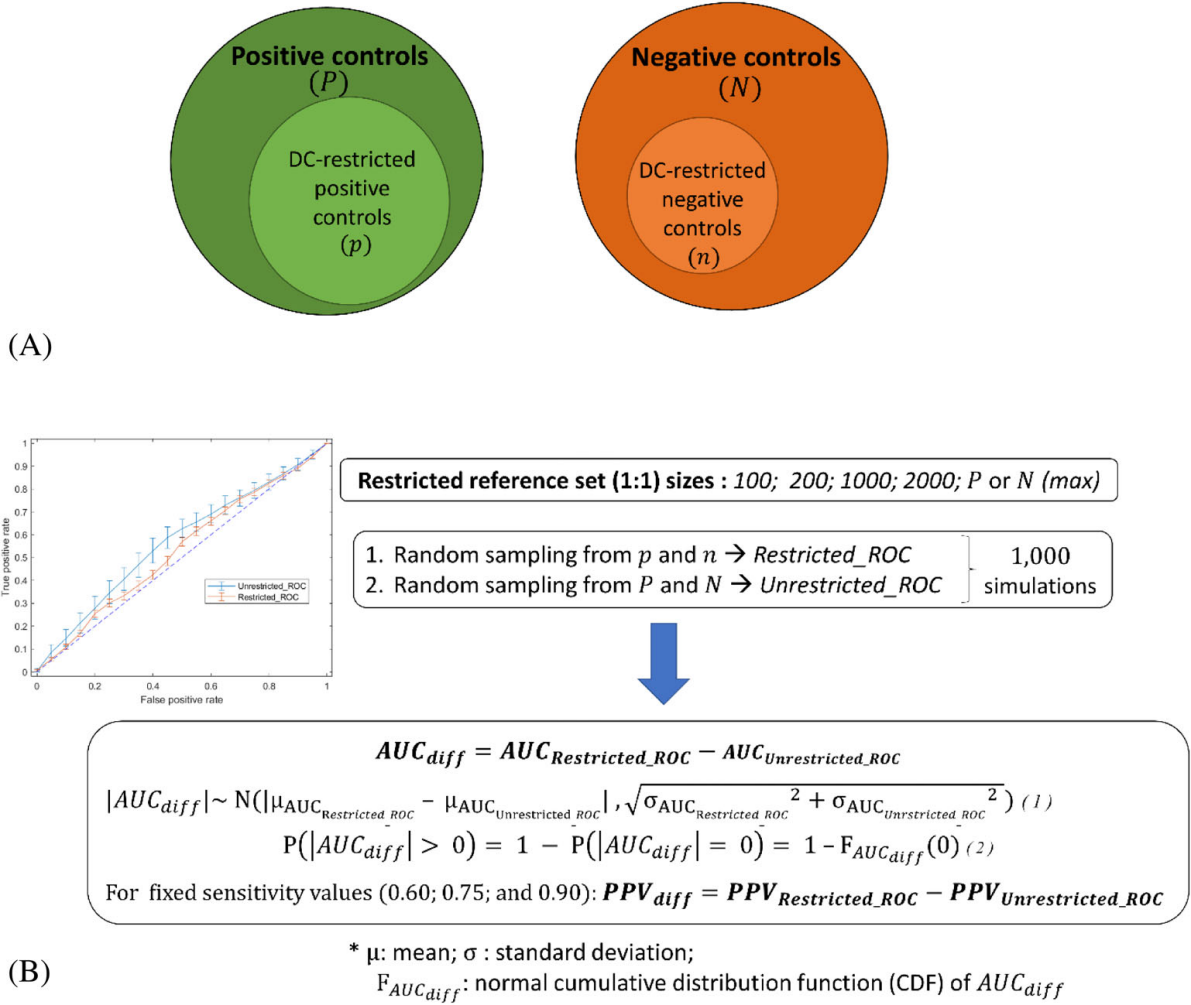
(A) Initial positive and negative control sets (P and N) and their respective restricted subsets (DC‐restricted, p and n) when applying a design criterion; (B) Simulation workflow for the differences in AUC (${\mathrm{AUC}}_{\text{diff}}$) and PPV (${\mathrm{PPV}}_{\text{diff}}$) when considering the specified design criterion.

Figure 1 illustrates the simulation workflow for the calculation of differences in AUC scores and PPV when considering the various design criteria.

## 3 RESULTS

**FIGURE 2:**
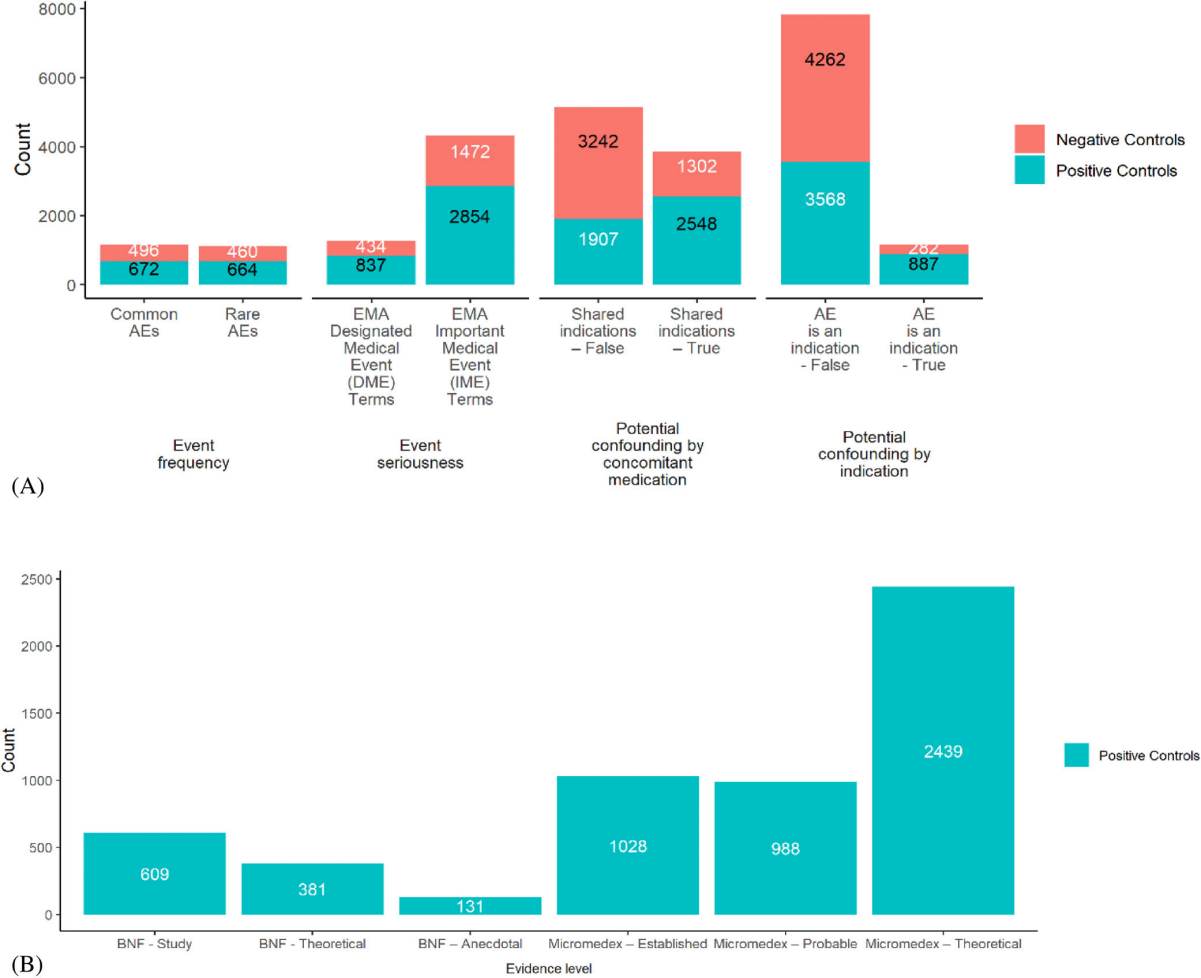
(A) Number of positive and negative controls from the PT Reference Set for each of the different design criteria when the restricted subsets contained both control types. The maximum number of controls considered from each type to form simulated reference sets ($N_{\max}$) is denoted with white color in the respective bar; (B) Number of PT Reference Set positive controls for the *Evidence level* design criteria, where the restriction could not be applied to negative controls.

**FIGURE 3:**
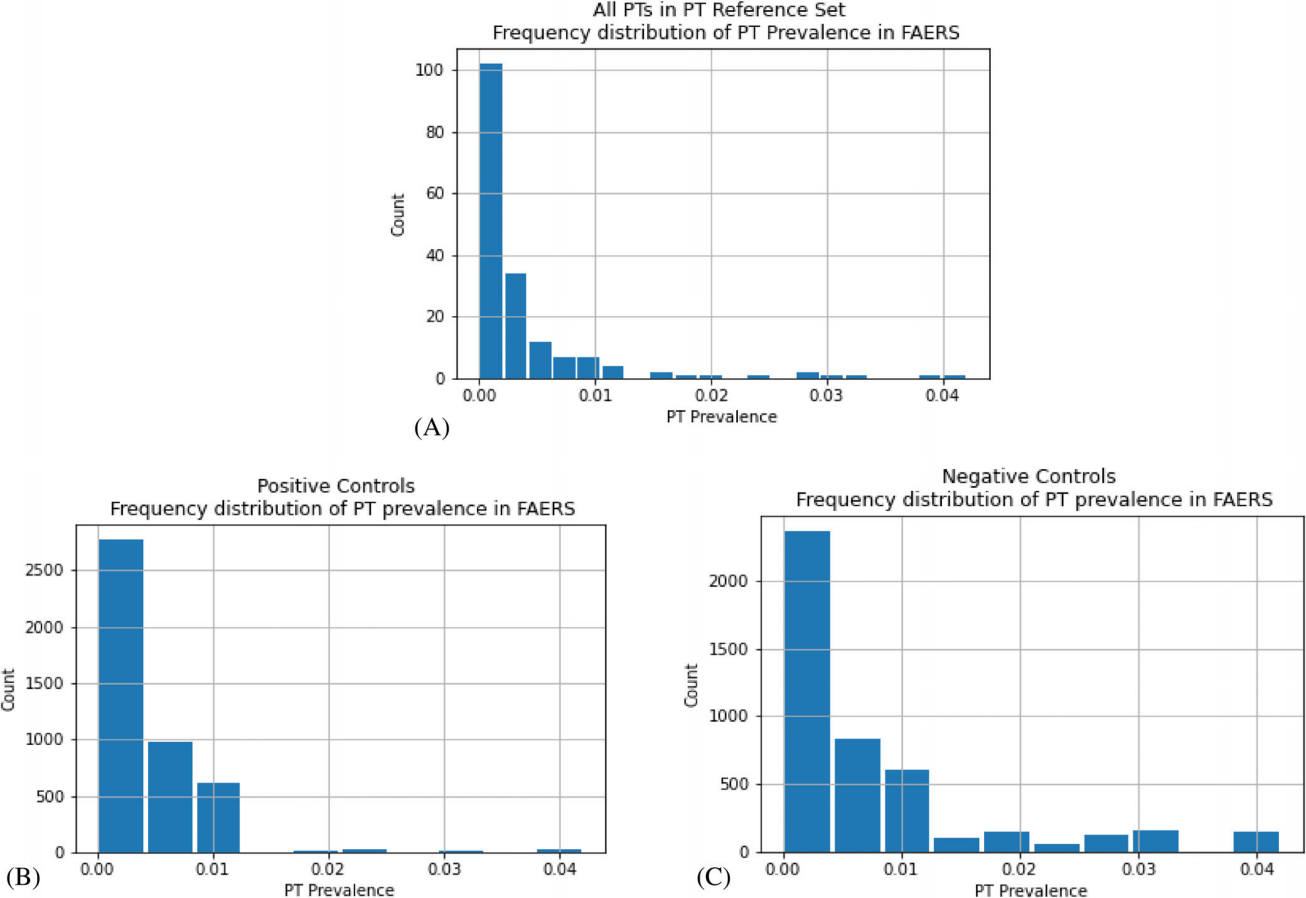
Frequency distribution of PT prevalence in FAERS for: (A) the set of unique PTs in the PT Reference Set; (B) PTs contained in the PT Reference Set positive controls; and (C) PTs contained in the PT Reference Set negative controls.

**FIGURE 4:**
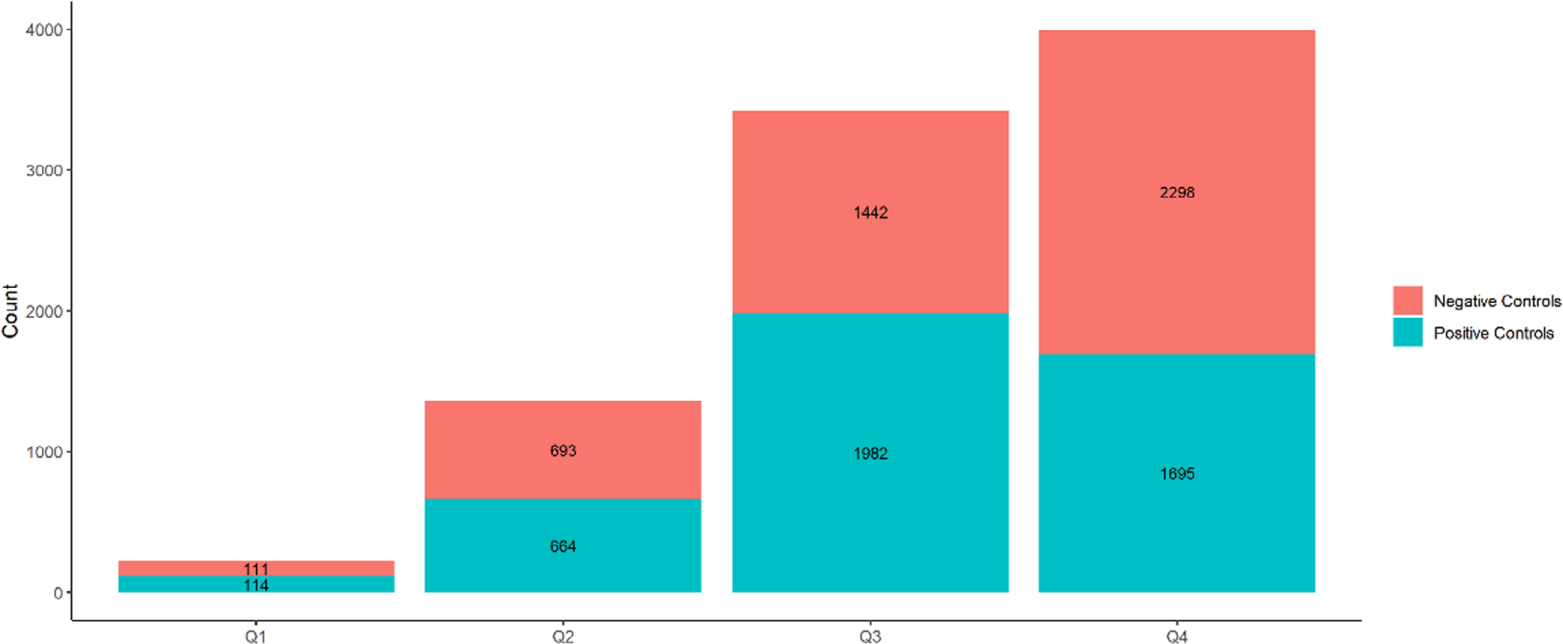
Number of positive and negative controls for groups Q1–Q4 that were formed using PT prevalence quartile binning, with Q1 containing the controls with the lowest prevalence and Q4 the highest one.

Τhe total number of positive and negative controls when applying each of the design criteria to the PT Reference Set is presented in Figure 2. In cases where restricted subsets contained both positive and negative controls (Figure 2A), the maximum number of controls considered from each type (i.e., positive or negative) to form simulated reference sets ($N_{\max}$) is denoted with white color in the respective bar. For the design criteria under the Evidence level category, where the restriction was only applied to positive controls (Figure 2B), $N_{\max}$ was defined as the total number of positive controls in the respective restricted subsets. Apart from two cases (i.e., Shared indications*—*False and AE is an indication*—*False), positive controls outnumbered negative controls in the restricted subsets. The simulated reference sets varied in size, with $N_{\max}$ ranging from 131 to 3568. Hence, more than 250 positive and negative controls were considered for every design criterion. For the MC Reference Set, the restricted subsets were smaller in size (Table S1). Three design criteria (BNF—Anecdotal, BNF—Theoretical, and AE is an indication—True) were not tested with this reference set, as their $N_{\max}$ was less than or equal to 100. Figure 3 provides the frequency distribution of PT prevalence in: (a) the set of unique PTs in the PT Reference Set; (b) PT Reference Set positive controls; and (c) PT Reference Set negative controls. The right‐tailed distribution of unique PTs in CRESCENDDI shows that the data set was populated with less common PTs, with only a small number of them having a prevalence over 0.01 in FAERS. Similar trends were present in the curves of the positive and negative controls, with the latter consisting of more cases with a higher PT prevalence in FAERS. The 1st, 2nd, and 3rd quartiles for the PT prevalence were 0.000343, 0.00135, and 0.00410, respectively. The total number of positive and negative controls for each group formed using PT prevalence quartile binning is shown in Figure 4. Group Q3 contained the largest volume in the case of positive controls, with Group Q1 and Group Q2 being considerably smaller, while negative controls showed an increasing trend while moving to groups of higher PT prevalence.

The MedDRA granularity affected the SDA performance metrics in different ways (Table 3). Omega and IntSS performed worse at the MC level as opposed to the PT level, with their mean AUC score dropping by 0.0605 and 0.0489, respectively. For Omega, there was a statistically significant decrease in the AUC between the PT and MC level evaluations. In the case of delta_add, the mean AUC slightly increased (0.0311) when considering the MC level, however without outperforming Omega.

**TABLE 3:** Statistics related to the performance evaluation of three SDAs for DDIs using matched controls from the PT Reference Set and MC Reference Set.

SDA	PT Reference Set AUC (95% CI)	MC Reference Set AUC (95% CI)
Omega	0.6011 (0.5704, 0.6317)	0.5406 (0.5150, 0.5662)
delta_add	0.4645 (0.4408, 0.4882)	0.4956 (0.4721, 0.5191)
IntSS	0.5374 (0.5100, 0.5648)	0.4885 (0.4654, 0.5117)

**FIGURE 5:**
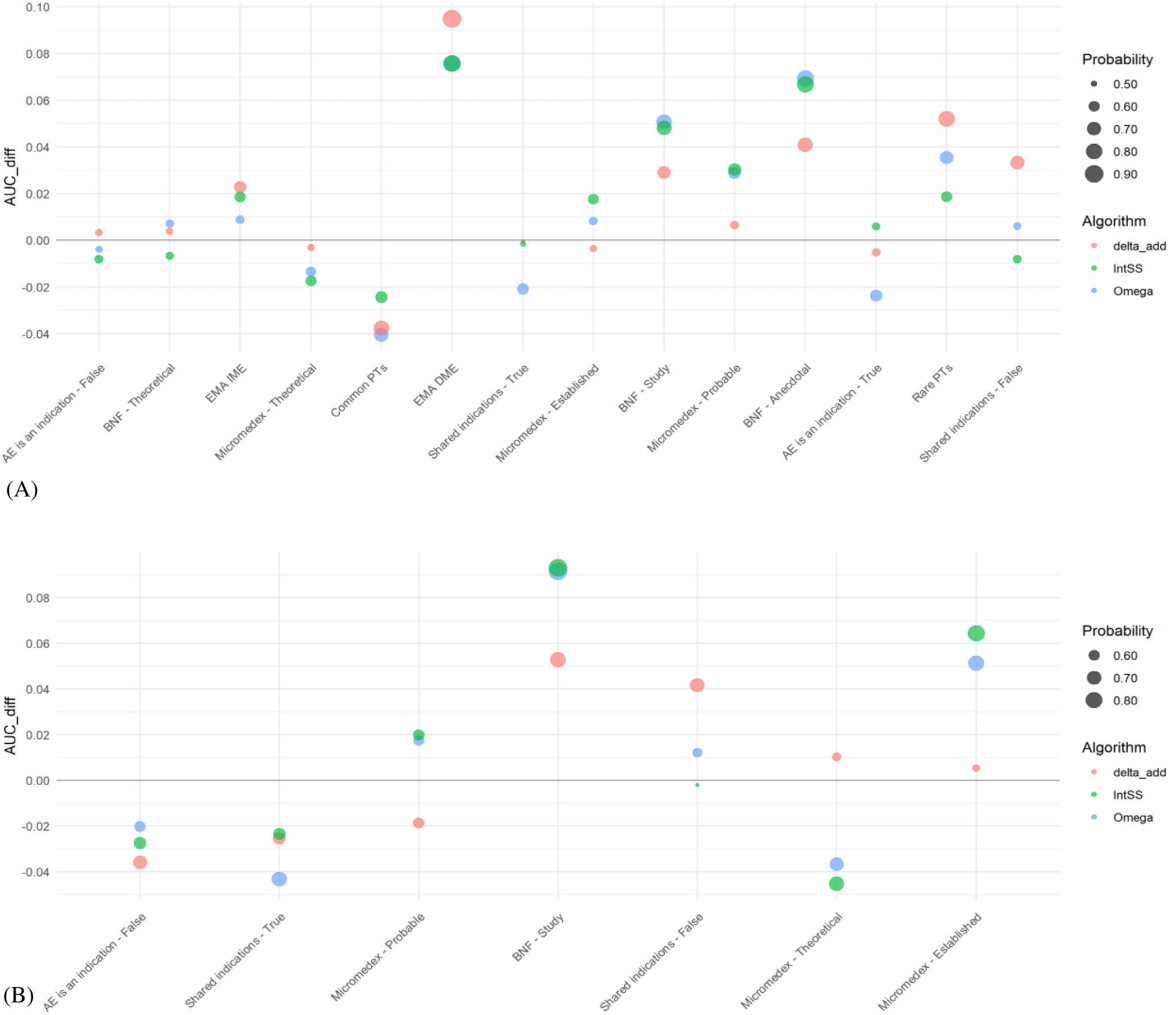
$\;{\mathrm{AUC}}_{\text{diff}}$ for a fixed restricted reference set size of 100 for: (A) the PT Reference Set; (B) the MC Reference Set. Design criteria are ordered by increasing range of ${\mathrm{AUC}}_{\text{diff}}$ values among the three SDAs. The dot size represents the probability of the estimated score, ${\mathrm{AUC}}_{\text{diff}}$, being non‐zero.

By plotting ${\mathrm{AUC}}_{\text{diff}}$ for a fixed constrained reference set size of 100 and ordering design criteria by increasing range of ${\mathrm{AUC}}_{\text{diff}}$ values among the three SDAs (Figures 5 and S1), points that lie above the x‐axis signify positive estimates for ${\mathrm{AUC}}_{\text{diff}}$, meaning that the design criterion had a positive effect on the calculated AUC. Conversely, points below the x‐axis were associated with a negative effect on the AUC when the specific design criterion was applied to constrain the reference set. Also, for the different sizes of restricted reference sets using the PT Reference Set and the MC Reference Set, ${\mathrm{AUC}}_{\text{diff}}$ value estimates and associated probabilities of a non‐zero ${\mathrm{AUC}}_{\text{diff}}$ estimate were plotted (Figures S2 and S3). With the PT Reference Set, the largest ${\mathrm{AUC}}_{\text{diff}}$ values were associated with the EMA Designated Medical Event Terms criterion (between 0.071 and 0.095), while Common PTs resulted in negative values in the range of −0.041 to −0.021 for the ${\mathrm{AUC}}_{\text{diff}}$ measure for all SDAs. In the case of the MC Reference Set, BNF—Study had the largest positive impact on all ${\mathrm{AUC}}_{\text{diff}}$ values (between 0.098 and 0.051), while negative ${\mathrm{AUC}}_{\text{diff}}$ values derived from Shared indications—True and AE is an indication—False (up to −0.043). Some design criteria affected the performance evaluation of all three SDAs in a similar way and level of magnitude (e.g., BNF—Anecdotal, BNF—Study), while others (e.g., Shared indication—False) seemed to have opposing and different in size effects on AUC estimates.

Tables S2 and S3 report the ${\mathrm{PPV}}_{\text{diff}}$ estimates (with 95% CIs) for the different design criteria, and a fixed reference set size of 100, for the PT Reference Set and MC Reference Set, respectively. For both reference sets and asensitivity equal to 0.60, some design criteria affected PPV in opposing ways among the different SDAs. For example, Shared indications—False resulted in negative ${\mathrm{PPV}}_{\text{diff}}$ estimates for Omega and IntSS (in the range between −0.029 and −0.021) as opposed to positive ones for delta_add (around 0.051). For other design criteria (i.e., BNF—Study and EMA—Designated Medical Events), ${\mathrm{PPV}}_{\text{diff}}$ estimates were positive across the different sensitivity values for all three SDAs. For a sensitivity value of 0.90, ${\mathrm{PPV}}_{\text{diff}}$ for the different design criteria were close to zero in all cases (values between 0.029 and −0.009).

With the PT Reference Set, we identified three main categories:

- Positive ${\mathrm{AUC}}_{\text{diff}}$ values
  - BNF—Anecdotal
  - EMA IME Terms
  - BNF—Study
  - Micromedex—Probable
  - EMA DME Terms
  - Rare PTs
- Negative ${\mathrm{AUC}}_{\text{diff}}$ values
  - Common PTs
  - Micromedex—Theoretical
- Mixed effect on ${\mathrm{AUC}}_{\text{diff}}$ values
  - AE is an indication‐False
  - AE is an indication‐True
  - Micromedex—Established
  - BNF—Theoretical
  - Only drug pairs that share at least one indication are included
  - Drug pairs that share at least one indication are excluded

With the MC Reference Set study, Omega and IntSS were affected in a similar way by the different design criteria. BNF–Study and Micromedex—Established had a positive impact on the target measure for all SDAs, while excluding AEs related to drugs' indications (AE is an indication–False) or only considering drug pairs with shared indications as controls (Shared indications–True) negatively affected the SDA performance in all cases.

**FIGURE 6:**
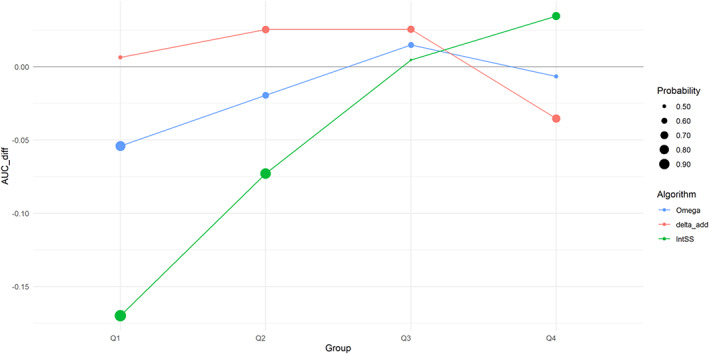
${\mathrm{AUC}}_{\text{diff}}$ values for Groups Q1–Q4 relevant to PT prevalence. The dot size represents the probability of the estimated score, ${\mathrm{AUC}}_{\text{diff}}$, being non‐zero.

In terms of PT prevalence (Figure 6), there was a similar trend for Groups Q1–Q3, with ${\mathrm{AUC}}_{\text{diff}}$ metric increasing for all algorithms as we moved to more common PTs. However, this relationship appears to be reversed in Group Q4, which contains the most frequent PTs in FAERS from the original data set, for Omega and delta_add, showing a negative impact on their AUC.

## 4 DISCUSSION

This study provides a systematic evaluation of the impact of multiple design criteria for reference sets on the comparative assessment of signal detection methodologies of adverse DDIs in SRS data. Performance assessment of SDAs in pharmacovigilance has often relied on the generation of custom‐made reference sets that consider exclusion or inclusion criteria to define eligible controls. Thus, the motivation behind this research was to examine how different criteria could affect the evaluation, potentially altering the conclusions regarding which algorithms perform best.

Our study highlighted that the relative composition of reference sets might significantly impact the evaluation metrics. Some criteria affect the comparison of different methodologies, such as the restriction of controls to only include PTs from the EMA's designated medical event list. Other criteria that were thought to have a potential effect on the evaluation process (e.g., anecdotal evidence supporting a positive control) were not found to significantly change the observed difference in metrics among the methodologies, as all of them were influenced in a similar way (Figure 5). Moreover, we found that the size of the reference set did not have a considerable effect on the ${\mathrm{AUC}}_{\text{diff}}$, although the associated probability of that metric being non‐zero increased when considering larger sizes (Figures S1 and S2). Apart from the AUC, commonly applied sensitivity values were considered to identify the impact of design criteria on PPV. For most of the design criteria (e.g., EMA Designated Medical Events, Micromedex evidence categories), ${\mathrm{PPV}}_{\text{diff}}$ values were affected consistently with the ${\mathrm{AUC}}_{\text{diff}}$ estimates across the three different SDAs. For the highest sensitivity that was considered (0.90), the difference in PPV was in most cases neglectable.

Given the inability of SDAs to account for all potential confounding factors that are present in SRS data, each methodology might be impacted to a different extent by a confounder. At the same time, there might be cases where signals are triggered by those confounding factors. As an illustrative example, the majority of DDI signals identified using IntSS in the original research paper^27^ were composed of drug pairs that are usually given concomitantly (e.g., antibiotics).^27^ We therefore need to consider the selection of appropriate controls to avoid misinterpretation of signals triggered by confounding factors rather than true associations as well as adding biases to our evaluation by “favoring” some algorithms while penalizing others. On the other hand, by attempting to completely remove all potential sources of confounding in our evaluation sets, we are more likely to fail to demonstrate their utility in real‐life application, which should be determined by its ability to perform at a commensurate level when it is applied prospectively to identify novel signals in SRS databases.^14^, ^15^ Overall, this analysis advocates the utilization of large, to the extent possible, reference sets when it comes to comparative performance assessment, that are less likely to suffer from the overrepresentation of controls that make different SDAs behave in different ways due to confounding. Also, regarding novel reference sets, the decision to restrict the evaluation set using specific design criteria should be adequately supported.

A major concern about reference sets used for prospective signal detection in pharmacovigilance revolves around the validity of established (i.e., well‐known) positive controls to test the performance of algorithms. This aspect has been widely discussed in the literature.^14^, ^15^, ^34^ It has been acknowledged that the combination of established and emerging positive controls might be a better choice when we try to evaluate the prospective performance and compare different methodologies, because merely emerging positive controls (i.e., recently detected ADRs) cannot establish a reliable reference standard.^18^ Especially for DDIs, the establishment of reference sets by only using emerging positive controls turns out to be particularly challenging, as we would end up having a very limited number of controls to be able to quantitatively assess differences in the performance of the SDAs under comparison. A solution to this issue would be to perform a backdated analysis to detect the time point that a signal of a true positive association (positive control) was first highlighted, as proposed in previous studies.^35^ However, this backdated analysis was not possible in this study due to the lack of a time‐indexed reference set for DDIs. A previous study compared the performance of SDA algorithms for DDI surveillance between established and emerging positive controls, with Omega and delta_add showing increased specificity but diminished sensitivity in the latter case.^19^ In our analysis, the results related to the evidence level are consistent with what we would expect to see. In terms of theoretical DDIs, it is common for drug interaction compendia to extend the included DDIs to the drug class level, therefore covering drugs under the same drug class that sometimes, but not necessarily, have a similar interaction profile. Our results showed declining AUC values when considering theoretical DDIs (i.e., Micromedex–Theoretical) as opposed to improvements with established ones (i.e., BNF–Study and Micromedex–Established). On the other hand, all three examined methodologies demonstrated enhanced performance against anecdotal DDIs from BNF and probable DDIs from Micromedex. However, the former category represented only a small fraction of the overall positive cases contained in the PT Reference Set (2.94%).

In terms of event background prevalence, the simulation results suggest that, if we restricted the evaluation set to specific ranges of PT prevalence, the conclusions would change, that is, the sole choice of common PTs would have an inverse impact on the comparative evaluation as to rare AEs. We know that SRS data are predominantly used in the post‐marketing setting to spot rare adverse reactions that have not been revealed during clinical trials. However, the use of SRS data for the detection of DDIs can be considered a different scenario, given that clinical trial data are not sufficient to detect adverse reactions of drug combinations due to inherent limitations (e.g., patient recruitment processes that exclude people taking multiple medications). Hence, the detection of novel DDI‐related adverse reactions, even with a common background rate, in SRS data should be of special interest.

Disease‐related AEs are a challenging issue in the effort to generate signals using SRS data, as confounding by indication can occur. A previous study reported that around 5% of the total reports for any drug in FAERS mention a drug's indication as an adverse event.^36^ This might be related to poor reporting quality or intended to report a disease's exacerbations due to a drug. Our results support that the choice of excluding disease‐related AEs (i.e., AE is an indication—False) did not have a significant effect on the AUC across the SDAs with the PT Reference Set, while it decreased the performance of all SDAs with the MC Reference Set. On the other hand, Omega demonstrated deteriorated performance in the scenario of detecting controls with AEs that were drugs' indications at the same time (i.e., AE is an indication—True), while the other two SDAs did not seem to be substantially affected by this design criterion.

Event seriousness has been used to build reference sets and assess SDA performance, as it could be utilized to filter signals in real‐life pharmacovigilance settings.^23^, ^24^ Our study suggests that, by only considering “significant” events, bias is introduced to evaluating SDAs that could be potentially used in routine pharmacovigilance to detect a broader set of events. Also, given that DMEs are rare events (i.e., have low prevalence) with a high drug‐attributable risk, it is important to note that this category might have been confounded to an extent by other design criteria categories that were considered in our study, such as the event frequency.

Quantitative signal detection is only one aspect of the more complex framework before a safety signal is validated. In the case of adverse DDI surveillance, previous studies have considered triage filters alongside disproportionality analysis to direct preliminary signal assessment.^37^, ^38^ These filters might be less suitable depending on the type of DDI. For example, there are more filters relevant to pharmacokinetic DDIs (e.g., cytochrome P450 activity) as opposed to pharmacodynamics interactions. Although the clinical significance of the differences between SDAs that are reported in this study might be questioned, it is important to note that quantitative methods for adverse DDI surveillance remain way less mature compared to those for single‐drug safety surveillance, also considering the additional complexity that is inherent to DDIs. In this way, the potential impact on real‐world pharmacovigilance could not be refuted, as even small changes in the performance of an SDA might have a considerable impact on the number of generated signals that are captured for further evaluation, leading to either missed signals or large amounts of potential signals that need to be evaluated, thus increasing the manual effort needed. It is also important to note that the three SDAs that were included in our study are not implemented to the same extent in the real world. Omega and IntSS are two of the major methods that we understand to be used for routine pharmacovigilance screening for DDIs. delta_add is a less mature method that is described in the literature, for which, as far as we are aware, is not as widely used in practice.

Although this study provides a novel framework for studying how SDA performance may change by considering different criteria for eligibility of controls, there are some limitations worth mentioning. First, only a single test data set (i.e., FAERS) was utilized for the purposes of this study. Also, CRESCENDDI was the only reference set utilized to generate estimates of the impact on AUC, in the absence of another comprehensive data set that could be used as a comparative source. We acknowledge that, by modifying the CRESCENDDI data set to consider adverse events at the MC level, we ended up with a smaller reference set that only included controls that could be represented by event groups (e.g., angioedema). This can have an impact on the extrapolation of the results and conclusions drawn from our analysis when considering single PTs as opposed to event groups. Additionally, for the determination of hit versus miss, it is important to consider how the results calculated at the PT level can depict the signal generation at the MC level. For example, if one SDA signals polymorphous ventricular tachycardia and another one signals torsade des points at the PT level, they have both made the same classification in real‐world pharmacovigilance, as both would have triggered the same case review by a diligent pharmacovigilance organization. The performance of SDAs was only assessed using the default values provided in the original research papers describing those methods (e.g., tuning parameter for shrinkage, *a*, equal to 0.5 in the case of Omega). Finally, the aspect of unbalanced reference sets was not explored in this study (i.e., positive to negative control ratio different from 1:1), since previous studies in pharmacovigilance have evaluated SDAs using asymmetrical reference sets.^18^, ^24^, ^31^

## 5 CONCLUSIONS

This study revealed a varying impact of design criteria for reference sets on the performance metrics of three SDAs that are used for DDI post‐marketing surveillance. This analysis showcases that the design of reference sets should be performed carefully, as the comparison of SDA performance might be affected by the choices made when building a reference set and the decision to restrict the evaluation to specific controls. Also, it highlights the need to establish frameworks that can make use of large and disparate data sources to support the generation of open‐source, flexible benchmarks in pharmacovigilance. These benchmarks can not only ensure transparency and enable a fair evaluation of SDA performance, but also provide a strong foundation that promotes productive research in pharmacovigilance signal detection methodologies.

## AUTHOR CONTRIBUTIONS

Elpida Kontsioti, Simon Maskell, and Munir Pirmohamed contributed to the conception and design of the study. Material preparation, data collection and analysis were performed by Elpida Kontsioti. The first draft of the manuscript was written by Elpida Kontsioti and all authors commented on previous versions of the manuscript. All authors read and approved the final manuscript.

## FUNDING INFORMATION

This study was jointly funded by EPSRC (grant number EP/R51231X/1) and AstraZeneca.

## CONFLICT OF INTEREST STATEMENT

Elpida Kontsioti received PhD studentship that was jointly funded by AstraZeneca and the EPSRC. She is currently an employee of The Hyve BV. Munir Pirmohamed receives research funding from various organizations including the MRC and NIHR. He has also received partnership funding for the MRC Clinical Pharmacology Training Scheme (co‐funded by MRC and Roche, UCB, Eli Lilly and Novartis) and grant funding from Vistagen Therapeutics. He has also unrestricted educational grant support for the UK Pharmacogenetics and Stratified Medicine Network from Bristol‐Myers Squibb and UCB. He has developed an HLA genotyping panel with MC Diagnostics, but does not benefit financially from this. He is part of the IMI Consortium ARDAT (www.ardat.org). These funding sources were not utilized for this work. Simon Maskell declares that he has no conflict of interest.

## Supporting information


**Figure S1.** ${\mathrm{AUC}}_{\text{diff}}$ for a fixed restricted reference set size of 100 with 95% confidence intervals for: (a) the PT Reference Set; (b) the MC Reference Set. Design criteria are ordered by increasing range of ${\mathrm{AUC}}_{\text{diff}}$ values among the three signal detection algorithms.
**Figure S2.** ${\mathrm{AUC}}_{\text{diff}}$ values for the different design criteria, signal detection algorithms, and sizes of restricted reference set for the PT Reference Set. In cases where the number of available controls in the restricted subset using a design criterion was smaller than 2000, there are missing points in the respective graph. Points that lie above the x‐axis signify positive estimates for ${\mathrm{AUC}}_{\text{diff}}$ (i.e., the design criterion had a positive effect on the calculated area under the curve), while those below the x‐axis were associated with a negative effect of the design criterion on the area under the curve score. The dot size represents the probability of the estimated score, ${\mathrm{AUC}}_{\text{diff}}$, being non‐zero.
**Figure S3.** ${\mathrm{AUC}}_{\text{diff}}$ estimated values and associated probabilities of a non‐zero ${\mathrm{AUC}}_{\text{diff}}$ estimate for the different design criteria, signal detection algorithms, and sizes of restricted reference set for the MC Reference Set. In cases where the number of available controls in the restricted subset using a design criterion was smaller than 200, there are missing points in the respective graph. Points that lie above the x‐axis signify positive estimates for ${\mathrm{AUC}}_{\text{diff}}$ (i.e., the design criterion had a positive effect on the calculated area under the curve), while those below the x‐axis were associated with a negative effect of the design criterion on the area under the curve score. The dot size represents the probability of the estimated score, ${\mathrm{AUC}}_{\text{diff}}$, being non‐zero.
**Table S1.** Number of positive and negative controls from the MC Reference Set for each of the different design criteria. The maximum number of controls considered from each type to form simulated reference sets (N_max) is denoted in **bold**. The design criteria in red were not tested due to the small number of their restricted sets.
**Table S2.** ${\mathrm{PPV}}_{\text{diff}}$ values with 95% confidence intervals (CIs) for the different design criteria, and a fixed restricted reference set size of 100 using the PT Reference Set. Green color represents estimates with a CI range containing only positive values. Red color represents estimates with a CI range containing only negative values.
**Table S3.** ${\mathrm{PPV}}_{\text{diff}}$ values with 95% CIs for the different design criteria, and a fixed restricted reference set size of 100 using the MC Reference Set. Green color represents estimates with a CI range containing only positive values. Red color represents estimates with a CI range containing only negative values.

Appendix S1.

Appendix S2.

## Data Availability

The CRESCENDDI data set that supports the findings of this study is openly available in Figshare at https://doi.org/10.6084/m9.figshare.c.5481408.v1.
