# Supplementary material for: Exploring the impact of design criteria for reference sets on performance evaluation of signal detection algorithms: The case of drug–drug interactions
Source: Pharmacoepidemiol Drug Saf. 2023 Mar 26;32(8):832–44. doi: 10.1002/pds.5609 (PMC10947279; doi:10.1002/pds.5609)
Supplement: Supplementary file 1 — Figure S1. AUCdiff for a fixed restricted reference set size of 100 with 95% confidence intervals for: (a) the PT Reference Set; (b) the MC Reference Set. Design criteria are ordered by increasing range of AUCdiff values among the three signal detection algorithms. Figure S2. AUCdiff values for the different design criteria, signal detection algorithms, and sizes of restricted reference set for the PT Reference Set. In cases where the number of available controls in the restricted subset using a design criterion was smaller than 2000, there are missing points in the respective graph. Points that lie above the x‐axis signify positive estimates for AUCdiff (i.e., the design criterion had a positive effect on the calculated area under the curve), while those below the x‐axis were associated with a negative effect of the design criterion on the area under the curve score. The dot size represents the probability of the estimated score, AUCdiff, being non‐zero. Figure S3. AUCdiff estimated values and associated probabilities of a non‐zero AUCdiff estimate for the different design criteria, signal detection algorithms, and sizes of restricted reference set for the MC Reference Set. In cases where the number of available controls in the restricted subset using a design criterion was smaller than 200, there are missing points in the respective graph. Points that lie above the x‐axis signify positive estimates for AUCdiff (i.e., the design criterion had a positive effect on the calculated area under the curve), while those below the x‐axis were associated with a negative effect of the design criterion on the area under the curve score. The dot size represents the probability of the estimated score, AUCdiff, being non‐zero. Table S1. Number of positive and negative controls from the MC Reference Set for each of the different design criteria. The maximum number of controls considered from each type to form simulated reference sets (N_max) is denoted in bold. The design criteria [file PDS-32-832-s003.docx]

**Supplementary Information**

**
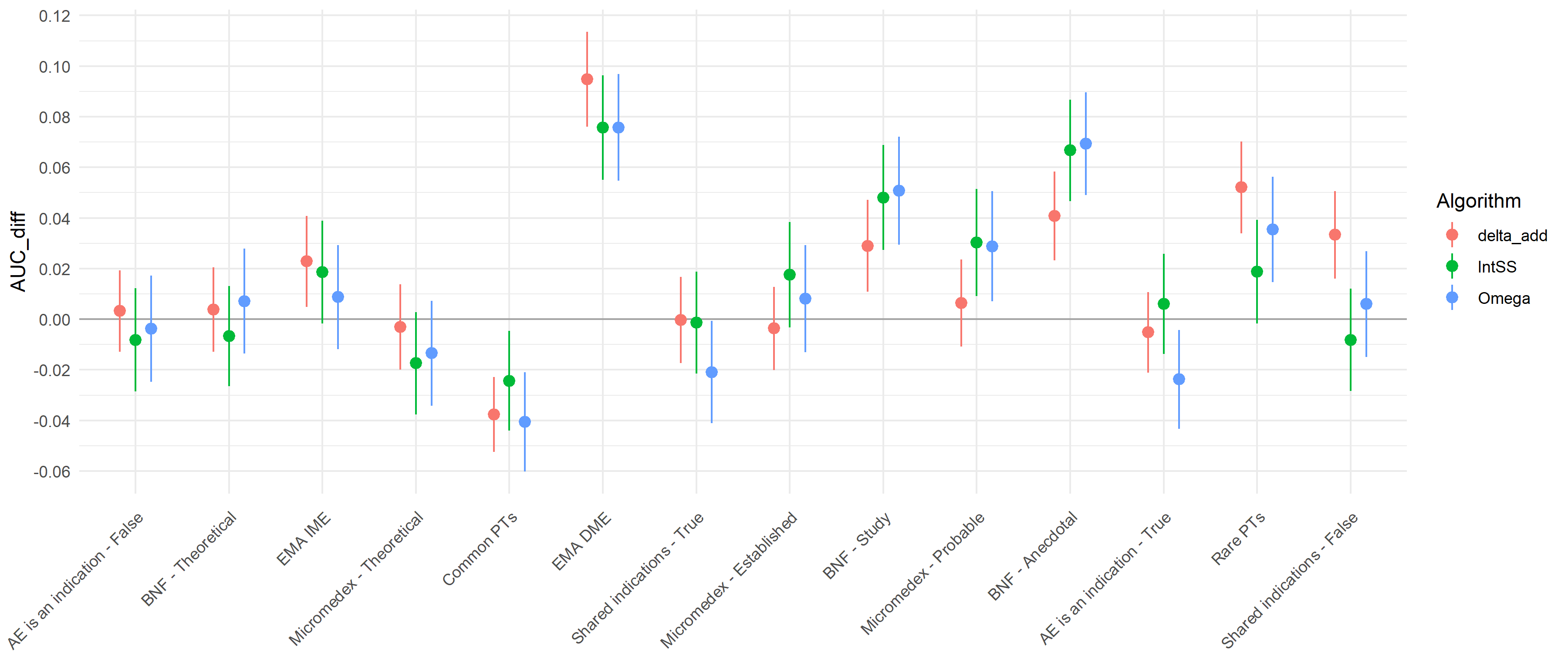
**

(a)

**
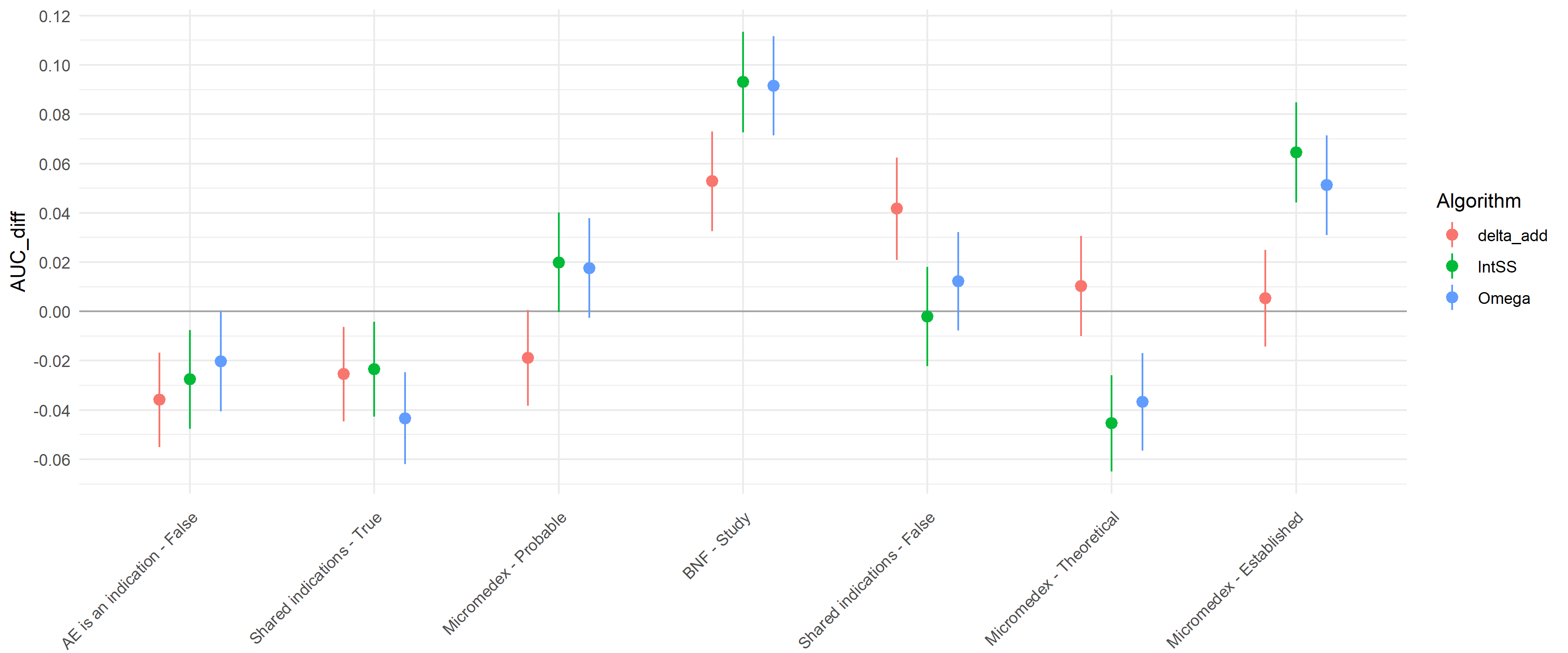
**

(b)

**Figure S1.** ${AUC}_{diff}$ for a fixed restricted reference set size of 100 with 95% confidence intervals for: (a) the ***PT Reference Set***; (b) the ***MC Reference Set***. Design criteria are ordered by increasing range of ${AUC}_{diff}$ values among the three signal detection algorithms.


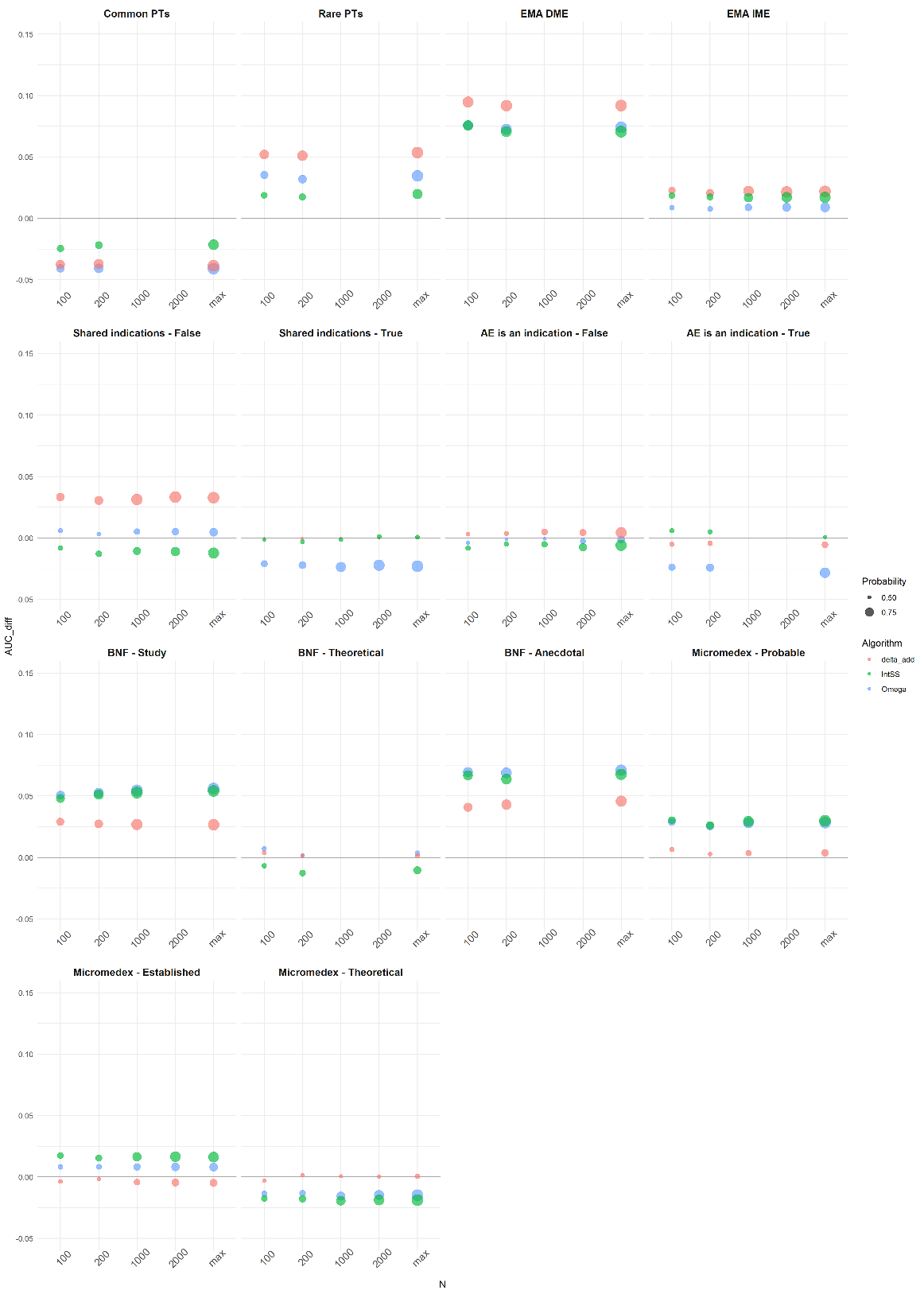


**Figure S2.** ${AUC}_{diff}$ values for the different design criteria, signal detection alogirithms, and sizes of restricted reference set for the ***PT Reference Set***. In cases where the number of available controls in the restricted subset using a design criterion was smaller than 2,000, there are missing points in the respective graph. Points that lie above the x-axis signify positive estimates for ${AUC}_{diff}$ (i.e., the design criterion had a positive effect on the calculated area under the curve), while those below the x-axis were associated with negative effect of the design criterion on the area under the curve score. The dot size represents the probability of the estimated score, ${AUC}_{diff}$, being non-zero.


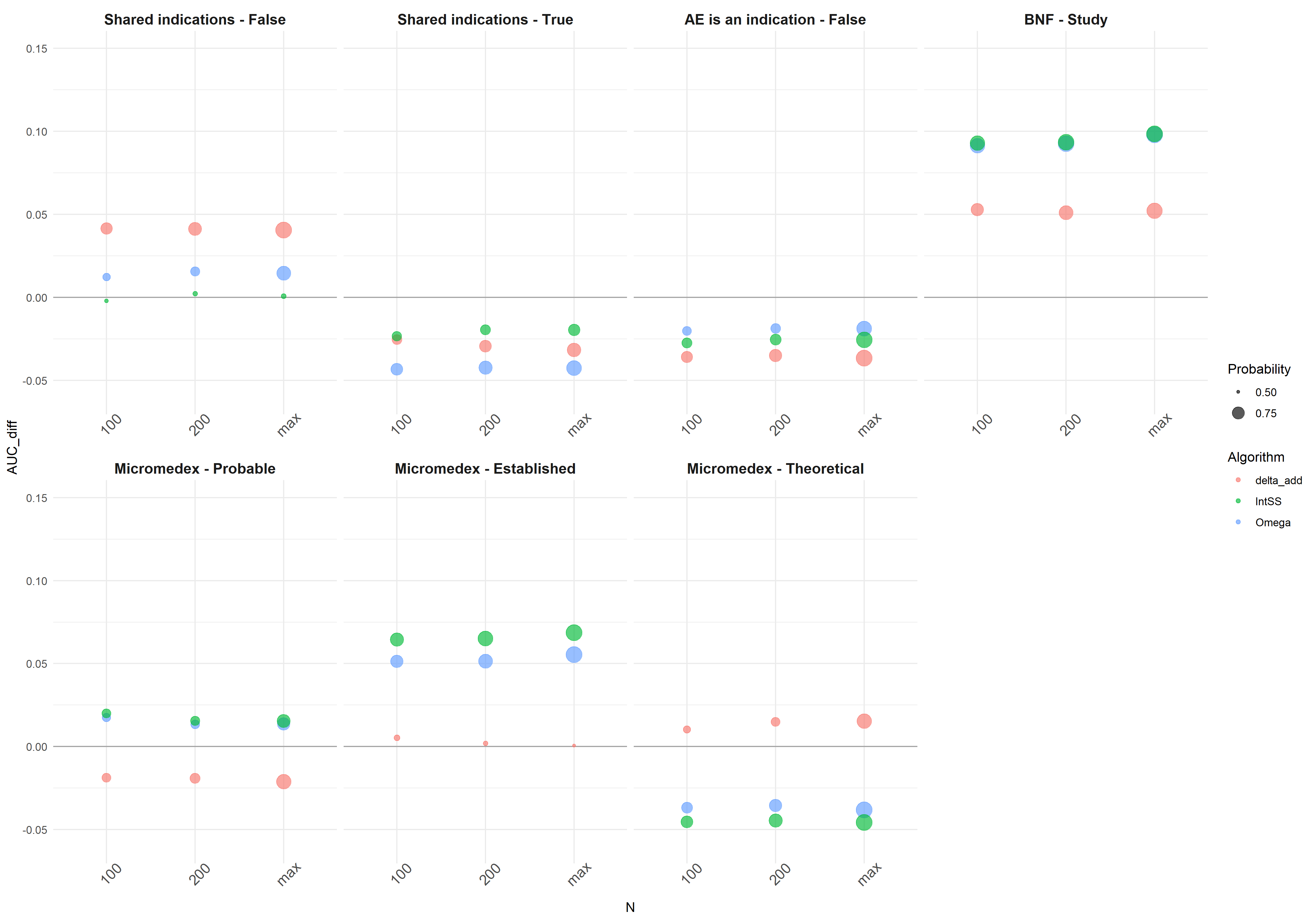
**Figure S3.** ${AUC}_{diff}$ estimated values and associated probabilities of a non-zero ${AUC}_{diff}$ estimate for the different design criteria, signal detection algorithms, and sizes of restricted reference set for the ***MC Reference Set***. In cases where the number of available controls in the restricted subset using a design criterion was smaller than 200, there are missing points in the respective graph. Points that lie above the x-axis signify positive estimates for ${AUC}_{diff}$ (i.e., the design criterion had a positive effect on the calculated area under the curve), while those below the x-axis were associated with negative effect of the design criterion on the area under the curve score. The dot size represents the probability of the estimated score, ${AUC}_{diff}$, being non-zero.

**Table S1.** Number of positive and negative controls from the ***MC Reference Set*** for each of the different design criteria. The maximum number of controls considered from each type to form simulated reference sets (N_max) is denoted with **bold**. The design criteria in red were not tested due to the small number of their restricted sets.

| **Design Criterion** | **N_pos** | **N_neg** |
| --- | --- | --- |
| BNF - Study | **184** | N/A |
| BNF - Theoretical | 100 | N/A |
| BNF - Anecdotal | 66 | N/A |
| Micromedex - Established | **235** | N/A |
| Micromedex - Theoretical | **498** | N/A |
| Micromedex - Probable | **364** | N/A |
| AE is an indication - True | 248 | 97 |
| AE is an indication - False | 849 | **517** |
| Shared indications – True | 569 | **169** |
| Shared indications – False | 528 | **445** |

**Table S2.** ${PPV}_{diff}$ values with 95% confidence intervals (CIs) for the different design criteria, and a fixed restricted reference set size of 100 using the ***PT Reference Set***. Green colour represents estimates with a CI range containing only positive values. Red colour represents estimates with a CI range containing only negative values.

| Design Criterion (DC) | Algorithm | ${PPV}_{diff}$ (95% CI) | | |
| --- | --- | --- | --- | --- |
|  |  | **Sensitivity** | | |
|  |  | 0.60 | 0.75 | 0.90 |
| *Shared indications - True* | Omega | -0.0089 (-0.0294, 0.0115) | 0.0066 (-0.0096, 0.0228) | 0.0064 (-0.0015, 0.0144) |
|  | delta_add | -0.0122 (-0.0243, -0.0001) | -0.0075 (-0.0135, -0.0014) | -0.0039 (-0.0073, -0.0006) |
|  | IntSS | 0.0177 (-0.0004, 0.0357) | 0.0169 (0.0053, 0.0285) | 0.0059 (0.0013, 0.0104) |
| *Shared indications - False* | Omega | -0.0225 (-0.0443, -0.0007) | -0.0135 (-0.0288, 0.0019) | -0.003 (-0.0105, 0.0044) |
|  | delta_add | 0.029 (0.0141, 0.0439) | 0.0099 (0.0026, 0.0172) | 0.0039 (0.0002, 0.0077) |
|  | IntSS | -0.027 (-0.0436, -0.0104) | -0.0148 (-0.0242, -0.0054) | -0.004 (-0.0076, -0.0005) |
| *AE is an indication - True* | Omega | -0.0056 (-0.0261, 0.0148) | 0.0078 (-0.0083, 0.024) | 0.0007 (-0.0069, 0.0083) |
|  | delta_add | 0.0193 (0.0072, 0.0314) | 0.0216 (0.0144, 0.0288) | 0.009 (0.0043, 0.0137) |
|  | IntSS | 0.0358 (0.0174, 0.0542) | 0.0251 (0.013, 0.0372) | 0.003 (-0.0014, 0.0074) |
| *AE is an indication - False* | Omega | -0.0092 (-0.0311, 0.0128) | -0.0055 (-0.0213, 0.0103) | -0.0022 (-0.0095, 0.0052) |
|  | delta_add | 0.0021 (-0.01, 0.0143) | 0.0011 (-0.0052, 0.0074) | 0.0004 (-0.0033, 0.004) |
|  | IntSS | -0.0126 (-0.0301, 0.0048) | -0.005 (-0.0147, 0.0048) | -0.0007 (-0.0043, 0.003) |
| *EMA IME* | Omega | 0.0179 (-0.005, 0.0409) | 0.0131 (-0.004, 0.0303) | 0.0073 (-0.001, 0.0157) |
|  | delta_add | 0.0066 (-0.0074, 0.0206) | -0.0009 (-0.0071, 0.0054) | -0.0017 (-0.0052, 0.0017) |
|  | IntSS | 0.0263 (0.007, 0.0456) | 0.017 (0.0056, 0.0285) | 0.0032 (-0.0009, 0.0073) |
| *EMA DME* | Omega | 0.0545 (0.0316, 0.0775) | 0.0517 (0.0344, 0.0689) | 0.0292 (0.0195, 0.039) |
|  | delta_add | 0.0729 (0.0544, 0.0915) | 0.0255 (0.0157, 0.0352) | 0.0091 (0.005, 0.0133) |
|  | IntSS | 0.0573 (0.0361, 0.0785) | 0.0277 (0.0153, 0.04) | 0.0047 (0, 0.0094) |
| *BNF - Anecdotal* | Omega | 0.0252 (0.003, 0.0475) | 0.0097 (-0.0067, 0.0261) | 0.0041 (-0.0038, 0.012) |
|  | delta_add | 0.0084 (-0.0047, 0.0215) | 0.0012 (-0.0053, 0.0078) | -0.0004 (-0.0039, 0.003) |
|  | IntSS | 0.0312 (0.012, 0.0505) | -0.0014 (-0.0129, 0.0101) | -0.007 (-0.01, -0.0041) |

| Design Criterion (DC) | Algorithm | $\boldsymbol{PPV}_{\boldsymbol{diff}}$ (95% CI) | | |
| --- | --- | --- | --- | --- |
|  |  | **Sensitivity** | | |
|  |  | 0.60 | 0.75 | 0.90 |
| *BNF - Study* | Omega | 0.037 (0.0148, 0.0593) | 0.0358 (0.0187, 0.0529) | 0.0116 (0.0026, 0.0205) |
|  | delta_add | 0.025 (0.0101, 0.0399) | 0.0109 (0.0036, 0.0182) | 0.005 (0.001, 0.009) |
|  | IntSS | 0.0449 (0.0255, 0.0642) | 0.0312 (0.0196, 0.0428) | 0.0112 (0.0061, 0.0163) |
| *BNF - Theoretical* | Omega | 0.0013 (-0.0205, 0.023) | -0.0052 (-0.0213, 0.011) | -0.0015 (-0.0085, 0.0055) |
|  | delta_add | -0.0057 (-0.0177, 0.0064) | -0.0079 (-0.014, -0.0017) | -0.0029 (-0.0063, 0.0004) |
|  | IntSS | -0.0213 (-0.0377, -0.005) | -0.0096 (-0.0185, -0.0007) | -0.0029 (-0.0064, 0.0005) |
| *Micromedex - Established* | Omega | 0.0202 (-0.001, 0.0415) | 0.0117 (-0.0057, 0.029) | 0.0049 (-0.0032, 0.0129) |
|  | delta_add | 0.0077 (-0.0057, 0.0212) | 0.0025 (-0.0044, 0.0094) | 0.0022 (-0.0016, 0.006) |
|  | IntSS | 0.028 (0.0095, 0.0465) | 0.0254 (0.0138, 0.0371) | 0.0117 (0.0069, 0.0165) |
| *Micromedex - Theoretical* | Omega | -0.0159 (-0.0377, 0.0058) | -0.0076 (-0.0233, 0.008) | -0.0023 (-0.0096, 0.005) |
|  | delta_add | -0.0016 (-0.0136, 0.0104) | 0.001 (-0.0054, 0.0075) | 0.0007 (-0.003, 0.0043) |
|  | IntSS | -0.0172 (-0.0343, -0.0001) | -0.0105 (-0.0198, -0.0012) | -0.0026 (-0.0059, 0.0007) |
| *Micromedex - Probable* | Omega | 0.0274 (0.0047, 0.05) | 0.0148 (-0.0033, 0.0329) | -0.0013 (-0.0092, 0.0065) |
|  | delta_add | 0.0022 (-0.0109, 0.0152) | -0.0048 (-0.0111, 0.0015) | -0.0028 (-0.0062, 0.0006) |
|  | IntSS | 0.0188 (-0.0012, 0.0388) | 0.005 (-0.0063, 0.0164) | -0.0007 (-0.0045, 0.003) |
| *Common PTs* | Omega | -0.031 (-0.0507, -0.0114) | -0.0078 (-0.0227, 0.0072) | -0.0037 (-0.0111, 0.0036) |
|  | delta_add | 0.007 (-0.0046, 0.0186) | 0.0106 (0.0036, 0.0175) | 0.0048 (0.0009, 0.0088) |
|  | IntSS | -0.0076 (-0.0244, 0.0092) | 0.0049 (-0.0051, 0.0149) | 0.0021 (-0.0016, 0.0059) |
| *Rare PTs* | Omega | -0.0104 (-0.036, 0.0151) | -0.0182 (-0.0336, -0.0028) | -0.006 (-0.0128, 0.0009) |
|  | delta_add | 0.008 (-0.0066, 0.0225) | -0.0129 (-0.0194, -0.0063) | -0.0089 (-0.0117, -0.0062) |
|  | IntSS | -0.0266 (-0.0433, -0.0099) | -0.0137 (-0.0229, -0.0044) | -0.0037 (-0.0068, -0.0006) |

**Table S3.** ${PPV}_{diff}$ values with 95% CIs for the different design criteria, and a fixed restricted reference set size of 100 using the ***MC Reference Set***. Green colour represents estimates with a CI range containing only positive values. Red colour represents estimates with a CI range containing only negative values.

| Design Criterion (DC) | Algorithm | $\boldsymbol{PPV}_{\boldsymbol{diff}}$(95% CI) | | |
| --- | --- | --- | --- | --- |
|  |  | **Sensitivity** | | |
|  |  | 0.60 | 0.75 | 0.90 |
| *Shared indications - True* | Omega | -0.0073 (-0.0376, 0.0087) | 0.0067 (-0.0064, 0.0198) | 0.0011 (-0.0051, 0.0073) |
|  | delta_add | -0.0382 (-0.0685, -0.0127) | -0.0106 (-0.0149, 0.0005) | -0.0012 (-0.0056, 0.0032) |
|  | IntSS | 0.0066 (-0.008, 0.0212) | 0.0166 (0.0063, 0.0269) | 0.0085 (0.0041, 0.0128) |
| *Shared indications - False* | Omega | -0.0203 (-0.0375, -0.003) | -0.0158 (-0.0283, -0.0032) | -0.0017 (-0.0081, 0.0047) |
|  | delta_add | 0.0506 (0.029, 0.0722) | 0.0164 (0.0055, 0.0272) | 0.0021 (-0.0026, 0.0068) |
|  | IntSS | -0.0288 (-0.0436, -0.014) | -0.0209 (-0.03, -0.0118) | -0.0054 (-0.0086, -0.0021) |
| *AE is an indication - False* | Omega | -0.01 (-0.0274, 0.0073) | -0.0093 (-0.0232, 0.0046) | -0.0044 (-0.0107, 0.002) |
|  | delta_add | -0.0296 (-0.0459, -0.0133) | -0.0149 (-0.0231, -0.0067) | -0.0069 (-0.0108, -0.003) |
|  | IntSS | -0.0142 (-0.029, 0.0006) | -0.0067 (-0.0168, 0.0034) | 0.0004 (-0.0033, 0.0042) |
| *BNF - Study* | Omega | 0.0789 (0.0602, 0.0976) | 0.0621 (0.0448, 0.0793) | 0.0143 (0.0065, 0.0221) |
|  | delta_add | 0.0917 (0.0708, 0.1126) | 0.0417 (0.0276, 0.0558) | 0.0096 (0.0045, 0.0147) |
|  | IntSS | 0.0834 (0.066, 0.1008) | 0.0477 (0.0315, 0.0639) | 0.0082 (0.0039, 0.0125) |
| *Micromedex - Established* | Omega | 0.0404 (0.0216, 0.0592) | 0.0329 (0.0179, 0.048) | 0.0059 (-0.0012, 0.013) |
|  | delta_add | 0.0023 (-0.0156, 0.0202) | 0.0112 (0.002, 0.0203) | 0.0053 (0.0002, 0.0105) |
|  | IntSS | 0.0531 (0.0354, 0.0708) | 0.0424 (0.0311, 0.0537) | 0.0203 (0.0144, 0.0261) |
| *Micromedex - Theoretical* | Omega | -0.0269 (-0.0438, -0.0099) | -0.0212 (-0.0338, -0.0086) | -0.005 (-0.0114, 0.0014) |
|  | delta_add | 0.0101 (-0.0091, 0.0293) | 0.0043 (-0.0051, 0.0137) | 0.0024 (-0.0023, 0.0071) |
|  | IntSS | -0.0275 (-0.042, -0.0129) | -0.0182 (-0.0277, -0.0087) | -0.0039 (-0.0071, -0.0006) |
| *Micromedex - Probable* | Omega | 0.0105 (-0.0069, 0.0279) | 0.0148 (0.0005, 0.0292) | 0.0044 (-0.0029, 0.0116) |
|  | delta_add | -0.0183 (-0.0361, -0.0004) | -0.0111 (-0.0191, -0.003) | -0.004 (-0.0083, 0.0002) |
|  | IntSS | 0.014 (-0.0034, 0.0314) | 0.0037 (-0.0069, 0.0144) | 0.0015 (-0.0023, 0.0053) |
